# Crystal structure and Hirshfeld surface analysis of (*E*)-3-[(4-fluoro­benzyl­idene)amino]-5-phenyl­thia­zolidin-2-iminium bromide

**DOI:** 10.1107/S2056989019004973

**Published:** 2019-04-18

**Authors:** Ali N. Khalilov, Zeliha Atioğlu, Mehmet Akkurt, Gulnara Sh. Duruskari, Flavien A. A. Toze, Afet T. Huseynova

**Affiliations:** a Organic Chemistry Department, Baku State University, Z. Xalilov str. 23, Az 1148, Baku, Azerbaijan; b Department of Physics and Chemistry, "Composite Materials" Scientific Research Center, Azerbaijan State Economic University (UNEC), H. Aliyev str. 135, Az 1063, Baku, Azerbaijan; c İlke Education and Health Foundation, Cappadocia University, Cappadocia Vocational College, The Medical Imaging Techniques Program, 50420 Mustafapaşa, Ürgüp, Nevşehir, Turkey; d Department of Physics, Faculty of Sciences, Erciyes University, 38039 Kayseri, Turkey; e Department of Chemistry, Faculty of Sciences, University of Douala, PO Box 24157, Douala, Republic of Cameroon

**Keywords:** crystal structure, charge assisted hydrogen bonding, thia­zolidine ring, disorder, Hirshfeld surface analysis.

## Abstract

In the cation of the title salt, C_16_H_15_FN_3_S^+^·Br^−^, the phenyl ring is disordered over two sets of sites with a refined occupancy ratio of 0.503 (4):0.497 (4). The mean plane of the thia­zolidine ring makes dihedral angles of 13.51 (14), 48.6 (3) and 76.5 (3)° with the fluoro­phenyl ring and the major- and minor-disorder components of the phenyl ring, respectively. The central thia­zolidine ring adopts an envelope conformation. In the crystal, centrosymmetrically related cations and anions are linked into dimeric units *via* N—H⋯Br hydrogen bonds, which are further connected by weak C—H⋯Br hydrogen bonds into chains parallel to [110]. Hirshfeld surface analysis and two-dimensional fingerprint plots indicate that the most important contributions to the crystal packing are from H⋯H (44.3%), Br⋯H/H⋯Br (16.8%), C⋯H/H⋯C (13.9%), F⋯H/H⋯F (10.3%) and S⋯H/H⋯S (3.8%) inter­actions.

## Abstract 2

In the crystal of the title salt, cations and anions are linked by N–H⋯Br hydrogen bonds forming inversion-related dimers. The dimers are connected by weak C–H⋯Br hydrogen bonds into chains.

## Chemical context   

Noncovalent inter­actions, both inter­molecular and intra­molecular, occur in virtually every substance and play an important role in the synthesis, catalysis, design of materials and biological processes (Akbari *et al.*, 2017 ▸; Gurbanov *et al.*, 2018 ▸; Kopylovich *et al.*, 2011 ▸; Maharramov *et al.*, 2010 ▸; Mahmoudi *et al.*, 2018*a* ▸,*b* ▸,*c* ▸; Mahmudov *et al.*, 2011 ▸, 2013 ▸, 2014*a* ▸,*b* ▸, 2015 ▸, 2017*a* ▸,*b* ▸, 2019 ▸; Shixaliyev *et al.*, 2013 ▸, 2018 ▸). On the other hand, Schiff bases and related hydrazone ligands and their complexes have attracted attention over the past decades due to their potential biological, pharmacological and analytical applications (Kopylovich *et al.*, 2011 ▸; Mahmoudi *et al.*, 2018*a* ▸,*b* ▸,*c* ▸; Mahmudov *et al.*, 2013 ▸). Hetercyclic amines are also widely used in the synthesis of Schiff bases, which provide different kinds of noncovalent inter­actions. As a further study in this field, we report herein the crystal structure and Hirshfeld surface analysis of the title compound.
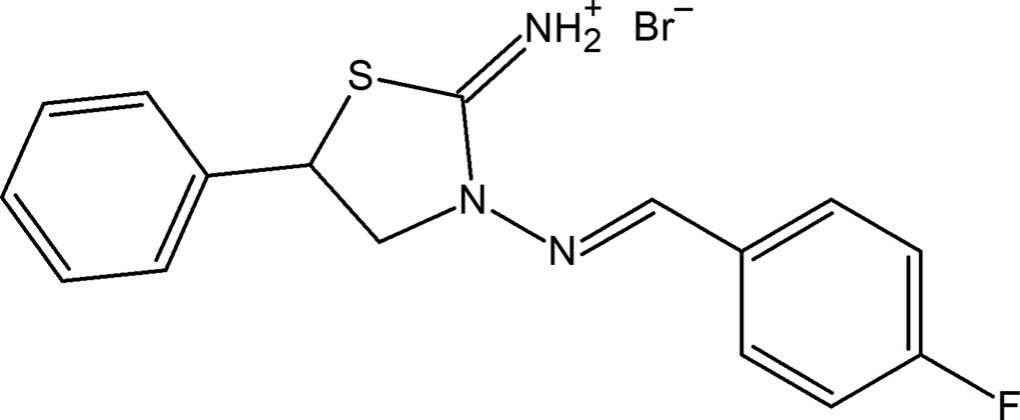


## Structural commentary   

**Figure 1:**
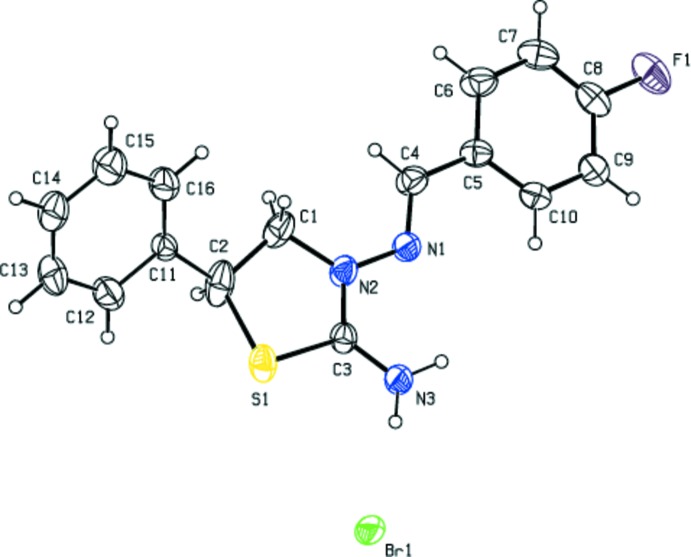
The mol­ecular structure of the title salt. Displacement ellipsoids are drawn at the 30% probability level. H atoms are shown as spheres of arbitrary radius. The minor-disorder component has been omitted for clarity

The thia­zolidine ring (S1/N2/C1–C3) in the cation of the title salt (Fig. 1 ▸) adopts an envelope conformation, with puckering parameters of *Q*(2) = 0.321 (3) Å and φ(2) = 43.3 (5)°. The mean plane of the thia­zolidine ring makes dihedral angles of 13.51 (14), 48.6 (3) and 76.5 (3)° with the fluoro­phenyl ring (C5–C10) and the major- and minor-disorder components (C11–C16 and C11′–C16′) of the phenyl ring, respectively. The N2—N1—C4—C5 bridge that links the thia­zolidine and 4-fluoro­phenyl rings has a torsion angle of −177.36 (19)°.

## Supra­molecular features and Hirshfeld surface analysis   

**Figure 2:**
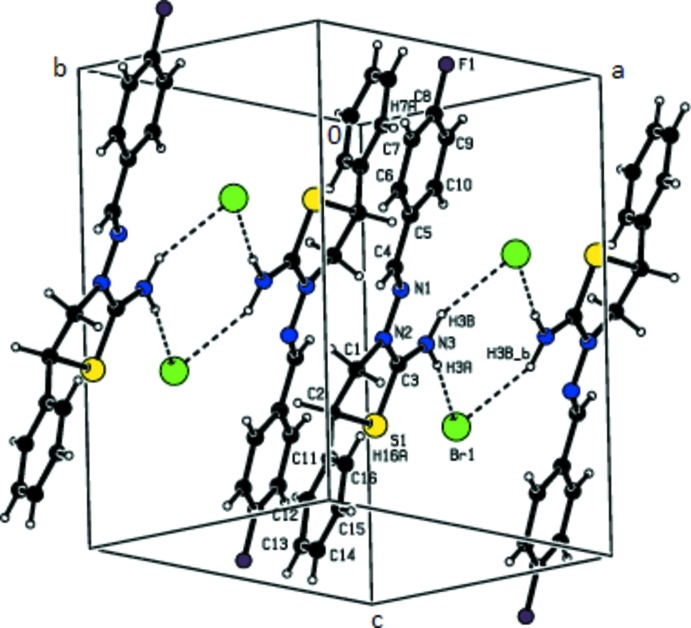
A view of the inter­molecular N—H⋯Br hydrogen bonds of the title salt in the unit cell. The minor-disorder component has been omitted for clarity

In the crystal, centrosymmetrically related cations and anions are linked *via* pairs of N—H⋯Br hydrogen bonds (Table 1 ▸) into dimeric units forming rings of 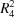
(8) graph-set motif (Fig. 2 ▸). The dimers are further connected by weak C—H⋯Br inter­actions to form chains running parallel to [110].

**Figure 3:**
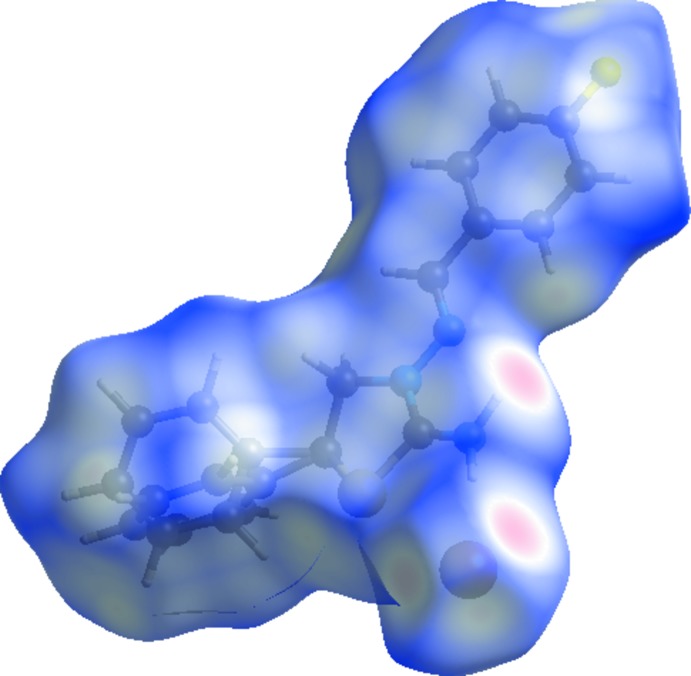
Hirshfeld surface of the title salt mapped with *d*_norm_.

Hirshfeld surface analysis was used to investigate the presence of hydrogen bonds and inter­molecular inter­actions in the crystal structure. The Hirshfeld surface analysis (Spackman & Jayatilaka, 2009 ▸) of the title salt was generated by *CrystalExplorer3.1* (Wolff *et al.*, 2012 ▸), and comprised *d*_norm_ surface plots and 2D fingerprint plots (Spackman & McKinnon, 2002 ▸). The plots of the Hirshfeld surface mapped over *d*_norm_ using a standard surface resolution with a fixed colour scale of −1.4747 (red) to 1.2166 a.u. (blue) is shown in Fig. 3 ▸. This plot was generated to qu­antify and visualize the inter­molecular inter­actions and to explain the observed crystal packing.

**Figure 4:**
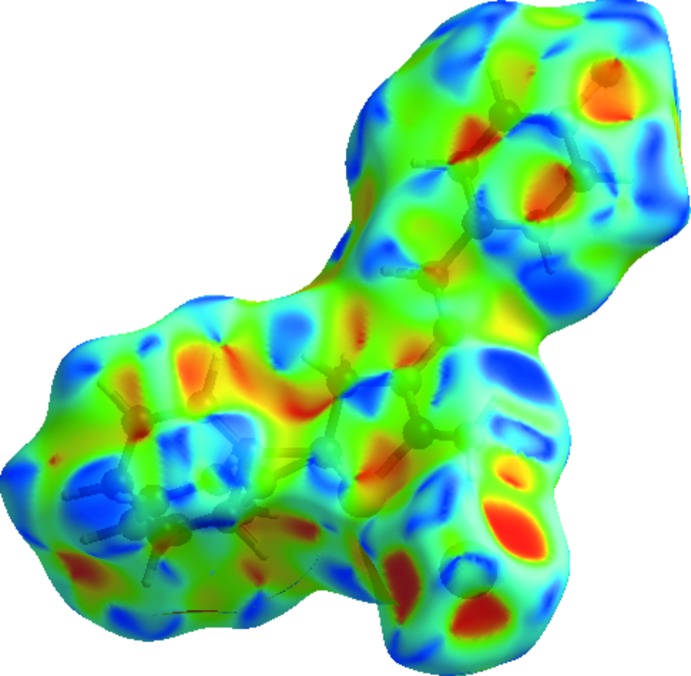
Hirshfeld surface of the title salt mapped with shape index.

The shape index of the Hirshfeld surface is a tool to visualize π–π stacking inter­actions by the presence of adjacent red and blue triangles; if there are no adjacent red and/or blue triangles, then there are no π–π inter­actions. Fig. 4 ▸ clearly suggest that there are no π–π inter­actions present in the title salt.

**Figure 5:**
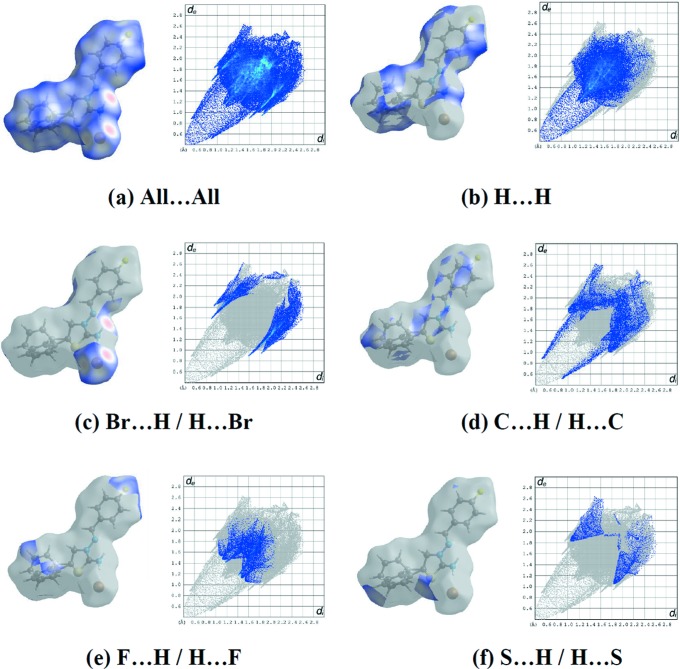
The 2D fingerprint plots of the title salt, showing (*a*) all inter­actions, and delineated into (*b*) H⋯H, (*c*) Br⋯H/H⋯Br, (*d*) C⋯H/H⋯C, (*e*) F⋯H/H⋯F and (*f*) S⋯H/H⋯S inter­actions [*d*_e_ and *d*_i_ represent the distances from a point on the Hirshfeld surface to the nearest atoms outside (external) and inside (inter­nal) the surface, respectively].

Fig. 5 ▸(*a*) shows the 2D fingerprint of the sum of the contacts contributing to the Hirshfeld surface represented in normal mode. These represent both the overall 2D fingerprint plots and those that represent H⋯H (44.3%), Br⋯H/H⋯Br (16.8%), C⋯H/H⋯C (13.9%), F⋯H/H⋯F (10.3%) and S⋯H/H⋯S (3.8%) contacts, respectively (Figs. 5 ▸ *b*–*f*). The most significant inter­molecular inter­actions are the H⋯H inter­actions (44.3%) (Fig. 6*b*). All the contributions to the Hirshfeld surface are given in Table 2 ▸.

### Database survey   

A search of the Cambridge Structural Database (CSD, Version 5.40, update of November 2018; Groom *et al.*, 2016 ▸) for 2-thia­zolidiniminium compounds gave seven hits, *viz*. UDELUN (Akkurt *et al.*, 2018 ▸), WILBIC (Marthi *et al.*, 1994 ▸), WILBOI (Marthi *et al.*, 1994 ▸), WILBOI01 (Marthi *et al.*, 1994 ▸), YITCEJ (Martem’yanova *et al.*, 1993*a* ▸), YITCAF (Martem’yanova *et al.*, 1993*b* ▸) and YOPLUK (Marthi *et al.*, 1995 ▸).

In the crystal of UDELUN (Akkurt *et al.*, 2018 ▸), C—H⋯Br and N—H⋯Br hydrogen bonds link the components into a three-dimensional network with the cations and anions stacked along the *b*-axis direction. Weak C—H⋯π inter­actions, which only involve the minor-disorder component of the ring, also contribute to the mol­ecular packing. In addition, there are also inversion-related Cl⋯Cl halogen bonds and C—Cl⋯π(ring) contacts.

In the remaining structures, the 3-N atom carries a C-substituent instead of an N-substituent, as found in the title compound. The first three crystal structures were determined for racemic (WILBIC; Marthi *et al.*, 1994 ▸) and two optically active samples (WILBOI and WILBOI01; Marthi *et al.*, 1994 ▸) of 3-(2′-chloro-2′-phenyl­eth­yl)-2-thia­zolidiniminium *p*-tolu­ene­sulfonate. In all three structures, the most disordered fragment of these mol­ecules is the asymmetric C atom and the Cl atom attached to it. The disorder of the cation in the racemate corresponds to the presence of both enanti­omers at each site in the ratio 0.821 (3):0.179 (3). The system of hydrogen bonds connecting two cations and two anions into 12-membered rings is identical in the racemic and in the optically active crystals. YITCEJ (Martem’yanova *et al.*, 1993*a* ▸) is a product of the inter­action of 2-amino-5-methyl­thia­zoline with methyl iodide, with alkyl­ation at the endocylic N atom, while YITCAF (Martem’yanova *et al.*, 1993*b* ▸) is a product of the reaction of 3-nitro-5-meth­oxy-, 3-nitro-5-chloro- and 3-bromo-5-nitro­salicyl­aldehyde with the heterocyclic base to form the salt-like complexes.

## Synthesis and crystallization   

To a solution of 3-amino-5-phenyl­thia­zolidin-2-iminium bro­mide (1 mmol) in ethanol (20 ml) was added 4-fluoro­benz­aldehyde (1 mmol). The mixture was refluxed for 2 h and then cooled. The reaction product precipitated from the reaction mixture as colourless single crystals, was collected by filtration and washed with cold acetone (yield 64%; m.p. 544–545 K). Analysis calculated (%) for C_16_H_15_BrFN_3_S: C 50.53, H 3.98, N 11.05; found: C 50.47, H 3.93, N 11.00. ^1^H NMR (300 MHz, DMSO-*d*_6_) : 4.56 (*k*, 1H, CH_2_, ^3^ *J*_H-H_ = 6.6 Hz), 4,87 (*t*, 1H, CH_2_, ^3^ *J*_H-H_ = 7.8 Hz), 5.60 (*t*, 1H, CH-Ar, 3*J*_H-H_ = 7.8 Hz), 7.32–8.16 (*m*, 9H, 9Ar-H), 8.45 (*s*, 1H, CH=), 10.37 (*s*, 2H, NH_2_). ^13^C NMR (75 MHz, DMSO-*d*_6_): 45.39, 55.97, 116.05, 127.81, 128.91, 129.13, 129.60, 131.05, 131.17, 137.55, 150.00, 167.89. MS (ESI), *m*/*z*: 300.36 [C_16_H_15_FN_3_S]^+^ and 79.88 Br^−^.

## Refinement details   

Crystal data, data collection and structure refinement details are summarized in Table 3 ▸. All H atoms were positioned geometrically and refined using a riding model, with N—H = 0.90 Å and C—H = 0.93–0.98 Å, and with *U*_iso_(H) = 1.2*U*_eq_(C,N). The phenyl ring in the cation is disordered over two sets of sites with an occupancy ratio of 0.503 (4):0.497 (4). Seven outliers (001; 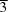
05; 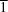
43; 010; 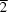
75; 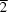,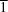,12; and 7
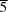
3) were omitted in the final cycles of refinement.

## Supplementary Material

Crystal structure: contains datablock(s) I. DOI: 10.1107/S2056989019004973/rz5254sup1.cif

Structure factors: contains datablock(s) I. DOI: 10.1107/S2056989019004973/rz5254Isup2.hkl

CCDC reference: 1909594

Additional supporting information:  crystallographic information; 3D view; checkCIF report

## Figures and Tables

**Table 1:** Hydrogen-bond geometry (Å, °)

*D*—H⋯*A*	*D*—H	H⋯*A*	*D*⋯*A*	*D*—H⋯*A*
N3—H3*A*⋯Br1	0.90	2.36	3.2557 (18)	172
N3—H3*B*⋯Br1^i^	0.90	2.55	3.3552 (18)	150
C4—H4*A*⋯Br1^ii^	0.93	2.99	3.726 (2)	137

Symmetry codes: (i) 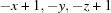; (ii) 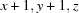.

**Table 2:** Percentage contributions of inter­atomic contacts to the Hirshfeld surface for the title salt

Contact	Percentage contribution
H⋯H	44.3
Br⋯H/H⋯Br	16.8
C⋯H/H⋯C	13.9
F⋯H/H⋯F	10.3
S⋯H/H⋯S	3.8
N⋯C/C⋯N	3.6
S⋯C/C⋯S	2.7
N⋯H/H⋯N	1.8
C⋯C	1.5
N⋯N	0.7
Br⋯C/C⋯Br	0.3
S⋯N/N⋯S	0.3
F⋯C/C⋯F	0.2

**Table 3:** Experimental details

Crystal data	
Chemical formula	C_16_H_15_FN_3_S^+^·Br^−^
*M*_r_	380.28
Crystal system, space group	Triclinic, *P* 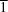
Temperature (K)	296
*a*, *b*, *c* (Å)	8.0599 (3), 8.6086 (4), 12.7608 (5)
α, β, γ (°)	96.548 (2), 92.518 (2), 111.065 (2)
*V* (Å^3^)	817.39 (6)
*Z*	2
Radiation type	Mo *K*α
μ (mm^−1^)	2.65
Crystal size (mm)	0.16 × 0.12 × 0.09

Data collection	
Diffractometer	Bruker APEXII CCD
Absorption correction	Multi-scan (*SADABS*; Bruker, 2003 ▸)
*T*_min_, *T*_max_	0.664, 0.782
No. of measured, independent and observed [*I* > 2σ(*I*)] reflections	12009, 3321, 2672
*R*_int_	0.025
(sin θ/λ)_max_ (Å^−1^)	0.627

Refinement	
*R*[*F* ^2^ > 2σ(*F* ^2^)], *wR*(*F* ^2^), *S*	0.029, 0.077, 1.04
No. of reflections	3321
No. of parameters	254
H-atom treatment	H-atom parameters constrained
Δρ_max_, Δρ_min_ (e Å^−3^)	0.37, −0.48

Computer programs: *APEX2* (Bruker, 2007 ▸), *SAINT* (Bruker, 2007 ▸), *SHELXS97* (Sheldrick, 2008 ▸), *SHELXL2014* (Sheldrick, 2015 ▸), *ORTEP-3 for Windows* (Farrugia, 2012 ▸) and *PLATON* (Spek, 2003 ▸).

## supplementary crystallographic information

### Crystal data

**Table:**

C_16_H_15_FN_3_S^+^·Br^−^	*Z* = 2
*M**_r_* = 380.28	*F*(000) = 384
Triclinic, *P*1	*D*_x_ = 1.545 Mg m^−^^3^
*a* = 8.0599 (3) Å	Mo *K*α radiation, λ = 0.71073 Å
*b* = 8.6086 (4) Å	Cell parameters from 5655 reflections
*c* = 12.7608 (5) Å	θ = 2.6–26.3°
α = 96.548 (2)°	µ = 2.65 mm^−^^1^
β = 92.518 (2)°	*T* = 296 K
γ = 111.065 (2)°	Plate, colorless
*V* = 817.39 (6) Å^3^	0.16 × 0.12 × 0.09 mm

### Data collection

**Table:**

Bruker APEXII CCD diffractometer	2672 reflections with *I* > 2σ(*I*)
φ and ω scans	*R*_int_ = 0.025
Absorption correction: multi-scan (SADABS; Bruker, 2003)	θ_max_ = 26.5°, θ_min_ = 2.7°
*T*_min_ = 0.664, *T*_max_ = 0.782	*h* = −10→7
12009 measured reflections	*k* = −10→10
3321 independent reflections	*l* = −14→15

### Refinement

**Table:**

Refinement on *F*^2^	0 restraints
Least-squares matrix: full	Hydrogen site location: mixed
*R*[*F*^2^ > 2σ(*F*^2^)] = 0.029	H-atom parameters constrained
*wR*(*F*^2^) = 0.077	*w* = 1/[σ^2^(*F*_o_^2^) + (0.0376*P*)^2^ + 0.2228*P*] where *P* = (*F*_o_^2^ + 2*F*_c_^2^)/3
*S* = 1.03	(Δ/σ)_max_ = 0.001
3321 reflections	Δρ_max_ = 0.37 e Å^−^^3^
254 parameters	Δρ_min_ = −0.48 e Å^−^^3^

### Special details

**Table:**

Geometry. All esds (except the esd in the dihedral angle between two l.s. planes) are estimated using the full covariance matrix. The cell esds are taken into account individually in the estimation of esds in distances, angles and torsion angles; correlations between esds in cell parameters are only used when they are defined by crystal symmetry. An approximate (isotropic) treatment of cell esds is used for estimating esds involving l.s. planes.

### Fractional atomic coordinates and isotropic or equivalent isotropic displacement parameters (Å^2^)

**Table:**

	*x*	*y*	*z*	*U*_iso_*/*U*_eq_	Occ. (<1)
Br1	0.28837 (4)	−0.06846 (3)	0.65093 (2)	0.07274 (12)	
S1	0.50535 (9)	0.40641 (8)	0.72511 (4)	0.06317 (17)	
F1	1.1652 (2)	0.6889 (3)	0.07197 (13)	0.0994 (6)	
N1	0.7677 (2)	0.5288 (2)	0.48291 (13)	0.0510 (4)	
C1	0.7491 (4)	0.6768 (3)	0.66453 (19)	0.0676 (7)	
H1A	0.765957	0.780305	0.635640	0.081*	
H1B	0.859205	0.688530	0.704392	0.081*	
N2	0.6981 (3)	0.5342 (2)	0.57951 (13)	0.0540 (4)	
C2	0.5974 (5)	0.6381 (4)	0.73431 (19)	0.0803 (9)	
H2A	0.506784	0.680819	0.710523	0.096*	
N3	0.5389 (3)	0.2468 (2)	0.54077 (14)	0.0564 (5)	
H3A	0.472588	0.153018	0.566419	0.068*	
H3B	0.598218	0.240158	0.483319	0.068*	
C3	0.5851 (3)	0.3881 (3)	0.60334 (16)	0.0489 (5)	
C4	0.8962 (3)	0.6588 (3)	0.46468 (18)	0.0583 (6)	
H4A	0.943400	0.751707	0.516854	0.070*	
C5	0.9692 (3)	0.6619 (3)	0.36204 (18)	0.0532 (5)	
C6	1.0980 (3)	0.8092 (3)	0.3405 (2)	0.0691 (7)	
H6A	1.139096	0.902914	0.392330	0.083*	
C7	1.1660 (4)	0.8188 (4)	0.2431 (2)	0.0767 (8)	
H7A	1.254083	0.916970	0.229104	0.092*	
C8	1.1011 (3)	0.6810 (4)	0.1683 (2)	0.0665 (7)	
C9	0.9756 (3)	0.5314 (3)	0.1862 (2)	0.0659 (6)	
H9A	0.935350	0.438646	0.133610	0.079*	
C10	0.9115 (3)	0.5229 (3)	0.28380 (19)	0.0592 (6)	
H10A	0.827792	0.422230	0.298028	0.071*	
C11	0.6893 (9)	0.7289 (7)	0.8505 (5)	0.0447 (13)	0.503 (4)
C12	0.5575 (7)	0.6983 (7)	0.9197 (5)	0.0691 (15)	0.503 (4)
H12A	0.439229	0.635805	0.894732	0.083*	0.503 (4)
C13	0.6019 (12)	0.7611 (13)	1.0269 (6)	0.074 (2)	0.503 (4)
H13A	0.513251	0.739287	1.073481	0.089*	0.503 (4)
C14	0.7747 (12)	0.8546 (10)	1.0639 (5)	0.0740 (16)	0.503 (4)
H14A	0.804349	0.898834	1.135130	0.089*	0.503 (4)
C15	0.9041 (8)	0.8822 (8)	0.9946 (4)	0.0811 (17)	0.503 (4)
H15A	1.022734	0.943608	1.019215	0.097*	0.503 (4)
C16	0.8600 (7)	0.8201 (7)	0.8893 (4)	0.0660 (14)	0.503 (4)
H16A	0.949586	0.841027	0.843322	0.079*	0.503 (4)
C11'	0.5982 (9)	0.7008 (8)	0.8494 (5)	0.0489 (14)	0.497 (4)
C12'	0.5168 (7)	0.8137 (6)	0.8805 (4)	0.0631 (13)	0.497 (4)
H12B	0.447047	0.841350	0.831371	0.076*	0.497 (4)
C13'	0.5408 (8)	0.8852 (7)	0.9865 (5)	0.0759 (18)	0.497 (4)
H13B	0.488252	0.962201	1.007970	0.091*	0.497 (4)
C14'	0.6390 (15)	0.8437 (12)	1.0570 (7)	0.079 (3)	0.497 (4)
H14B	0.655646	0.893516	1.127240	0.094*	0.497 (4)
C15'	0.7133 (14)	0.7321 (12)	1.0286 (5)	0.0843 (19)	0.497 (4)
H15B	0.779040	0.702274	1.078940	0.101*	0.497 (4)
C16'	0.6923 (8)	0.6598 (8)	0.9226 (5)	0.0793 (17)	0.497 (4)
H16B	0.744737	0.582013	0.902974	0.095*	0.497 (4)

### Atomic displacement parameters (Å^2^)

**Table:**

	*U*^11^	*U*^22^	*U*^33^	*U*^12^	*U*^13^	*U*^23^
Br1	0.0842 (2)	0.05494 (16)	0.05450 (16)	−0.00131 (12)	0.02403 (12)	−0.00748 (11)
S1	0.0911 (4)	0.0681 (4)	0.0419 (3)	0.0401 (3)	0.0203 (3)	0.0126 (3)
F1	0.1003 (12)	0.1287 (15)	0.0746 (10)	0.0375 (11)	0.0439 (9)	0.0375 (10)
N1	0.0588 (10)	0.0484 (10)	0.0428 (9)	0.0154 (9)	0.0093 (8)	0.0067 (8)
C1	0.1015 (19)	0.0518 (13)	0.0448 (12)	0.0257 (13)	−0.0005 (12)	−0.0010 (10)
N2	0.0706 (12)	0.0494 (10)	0.0385 (9)	0.0183 (9)	0.0088 (8)	0.0032 (8)
C2	0.145 (3)	0.0759 (18)	0.0422 (13)	0.0652 (19)	0.0157 (15)	0.0117 (12)
N3	0.0696 (11)	0.0478 (10)	0.0492 (10)	0.0162 (9)	0.0199 (9)	0.0087 (8)
C3	0.0593 (12)	0.0513 (12)	0.0400 (10)	0.0240 (10)	0.0079 (9)	0.0079 (9)
C4	0.0643 (14)	0.0516 (13)	0.0492 (12)	0.0108 (11)	0.0016 (10)	0.0044 (10)
C5	0.0504 (12)	0.0514 (12)	0.0535 (12)	0.0119 (10)	0.0028 (10)	0.0139 (10)
C6	0.0733 (16)	0.0558 (14)	0.0663 (15)	0.0074 (12)	0.0053 (13)	0.0159 (12)
C7	0.0708 (16)	0.0692 (17)	0.0845 (19)	0.0101 (13)	0.0177 (14)	0.0362 (16)
C8	0.0585 (14)	0.0891 (19)	0.0617 (15)	0.0311 (14)	0.0196 (12)	0.0303 (14)
C9	0.0575 (14)	0.0732 (16)	0.0636 (15)	0.0204 (12)	0.0141 (11)	0.0049 (12)
C10	0.0515 (12)	0.0560 (13)	0.0627 (14)	0.0092 (10)	0.0148 (11)	0.0103 (11)
C11	0.052 (3)	0.043 (3)	0.043 (3)	0.018 (3)	0.015 (3)	0.012 (2)
C12	0.060 (3)	0.084 (4)	0.059 (4)	0.021 (3)	0.014 (3)	0.005 (3)
C13	0.084 (6)	0.101 (6)	0.045 (4)	0.042 (5)	0.018 (4)	0.009 (4)
C14	0.100 (5)	0.083 (5)	0.042 (3)	0.038 (4)	0.006 (3)	0.001 (3)
C15	0.085 (4)	0.096 (4)	0.055 (3)	0.029 (3)	0.003 (3)	0.001 (3)
C16	0.066 (3)	0.077 (3)	0.049 (3)	0.020 (3)	0.011 (2)	0.003 (2)
C11'	0.051 (4)	0.053 (3)	0.036 (3)	0.009 (3)	0.016 (3)	0.007 (2)
C12'	0.066 (3)	0.058 (3)	0.065 (3)	0.019 (2)	0.009 (2)	0.020 (2)
C13'	0.092 (4)	0.057 (3)	0.083 (4)	0.031 (3)	0.041 (3)	0.006 (3)
C14'	0.095 (7)	0.073 (5)	0.042 (4)	0.002 (5)	0.013 (4)	−0.004 (3)
C15'	0.087 (5)	0.111 (7)	0.055 (4)	0.037 (5)	−0.011 (3)	0.012 (4)
C16'	0.084 (4)	0.096 (4)	0.072 (4)	0.053 (4)	0.008 (3)	0.002 (3)

### Geometric parameters (Å, º)

**Table:**

S1—C3	1.720 (2)	C9—C10	1.369 (3)
S1—C2	1.848 (3)	C9—H9A	0.9300
F1—C8	1.353 (3)	C10—H10A	0.9300
N1—C4	1.276 (3)	C11—C16	1.353 (8)
N1—N2	1.380 (2)	C11—C12	1.383 (7)
C1—N2	1.465 (3)	C12—C13	1.393 (10)
C1—C2	1.506 (4)	C12—H12A	0.9300
C1—H1A	0.9700	C13—C14	1.364 (14)
C1—H1B	0.9700	C13—H13A	0.9300
N2—C3	1.339 (3)	C14—C15	1.370 (9)
C2—C11'	1.505 (6)	C14—H14A	0.9300
C2—C11	1.607 (7)	C15—C16	1.370 (7)
C2—H2A	0.9800	C15—H15A	0.9300
N3—C3	1.297 (3)	C16—H16A	0.9300
N3—H3A	0.9001	C11'—C16'	1.333 (9)
N3—H3B	0.9000	C11'—C12'	1.389 (8)
C4—C5	1.458 (3)	C12'—C13'	1.394 (8)
C4—H4A	0.9300	C12'—H12B	0.9300
C5—C6	1.386 (3)	C13'—C14'	1.334 (12)
C5—C10	1.390 (3)	C13'—H13B	0.9300
C6—C7	1.379 (4)	C14'—C15'	1.329 (15)
C6—H6A	0.9300	C14'—H14B	0.9300
C7—C8	1.358 (4)	C15'—C16'	1.399 (9)
C7—H7A	0.9300	C15'—H15B	0.9300
C8—C9	1.373 (4)	C16'—H16B	0.9300
			
C3—S1—C2	90.65 (11)	C10—C9—H9A	121.0
C4—N1—N2	117.98 (19)	C8—C9—H9A	121.0
N2—C1—C2	105.8 (2)	C9—C10—C5	121.2 (2)
N2—C1—H1A	110.6	C9—C10—H10A	119.4
C2—C1—H1A	110.6	C5—C10—H10A	119.4
N2—C1—H1B	110.6	C16—C11—C12	118.7 (5)
C2—C1—H1B	110.6	C16—C11—C2	133.2 (5)
H1A—C1—H1B	108.7	C12—C11—C2	108.2 (5)
C3—N2—N1	116.37 (17)	C11—C12—C13	120.0 (6)
C3—N2—C1	115.67 (18)	C11—C12—H12A	120.0
N1—N2—C1	127.52 (19)	C13—C12—H12A	120.0
C11'—C2—C1	129.4 (4)	C14—C13—C12	120.3 (7)
C1—C2—C11	104.7 (3)	C14—C13—H13A	119.8
C11'—C2—S1	104.9 (3)	C12—C13—H13A	119.8
C1—C2—S1	105.04 (17)	C13—C14—C15	119.0 (6)
C11—C2—S1	112.7 (2)	C13—C14—H14A	120.5
C1—C2—H2A	111.4	C15—C14—H14A	120.5
C11—C2—H2A	111.4	C16—C15—C14	120.5 (6)
S1—C2—H2A	111.4	C16—C15—H15A	119.7
C3—N3—H3A	117.3	C14—C15—H15A	119.7
C3—N3—H3B	119.6	C11—C16—C15	121.5 (5)
H3A—N3—H3B	120.5	C11—C16—H16A	119.2
N3—C3—N2	123.59 (19)	C15—C16—H16A	119.2
N3—C3—S1	123.18 (17)	C16'—C11'—C12'	118.8 (6)
N2—C3—S1	113.23 (16)	C16'—C11'—C2	119.7 (5)
N1—C4—C5	120.0 (2)	C12'—C11'—C2	121.2 (5)
N1—C4—H4A	120.0	C11'—C12'—C13'	119.1 (5)
C5—C4—H4A	120.0	C11'—C12'—H12B	120.4
C6—C5—C10	118.6 (2)	C13'—C12'—H12B	120.4
C6—C5—C4	119.1 (2)	C14'—C13'—C12'	120.3 (6)
C10—C5—C4	122.3 (2)	C14'—C13'—H13B	119.9
C7—C6—C5	120.8 (3)	C12'—C13'—H13B	119.9
C7—C6—H6A	119.6	C15'—C14'—C13'	121.0 (8)
C5—C6—H6A	119.6	C15'—C14'—H14B	119.5
C8—C7—C6	118.3 (2)	C13'—C14'—H14B	119.5
C8—C7—H7A	120.9	C14'—C15'—C16'	119.7 (8)
C6—C7—H7A	120.9	C14'—C15'—H15B	120.1
F1—C8—C7	119.0 (2)	C16'—C15'—H15B	120.1
F1—C8—C9	117.9 (3)	C11'—C16'—C15'	121.0 (6)
C7—C8—C9	123.1 (2)	C11'—C16'—H16B	119.5
C10—C9—C8	118.0 (3)	C15'—C16'—H16B	119.5
			
C4—N1—N2—C3	−169.1 (2)	C6—C5—C10—C9	−2.0 (4)
C4—N1—N2—C1	3.0 (3)	C4—C5—C10—C9	176.7 (2)
C2—C1—N2—C3	−26.1 (3)	C1—C2—C11—C16	−1.1 (7)
C2—C1—N2—N1	161.8 (2)	S1—C2—C11—C16	112.5 (6)
N2—C1—C2—C11'	155.8 (4)	C1—C2—C11—C12	−179.8 (4)
N2—C1—C2—C11	150.2 (3)	S1—C2—C11—C12	−66.2 (5)
N2—C1—C2—S1	31.4 (2)	C16—C11—C12—C13	0.1 (10)
C3—S1—C2—C11'	−163.6 (3)	C2—C11—C12—C13	178.9 (6)
C3—S1—C2—C1	−24.92 (19)	C11—C12—C13—C14	0.8 (14)
C3—S1—C2—C11	−138.3 (3)	C12—C13—C14—C15	−1.5 (15)
N1—N2—C3—N3	−0.7 (3)	C13—C14—C15—C16	1.4 (12)
C1—N2—C3—N3	−173.7 (2)	C12—C11—C16—C15	−0.2 (9)
N1—N2—C3—S1	179.87 (14)	C2—C11—C16—C15	−178.7 (5)
C1—N2—C3—S1	6.8 (3)	C14—C15—C16—C11	−0.6 (10)
C2—S1—C3—N3	−168.1 (2)	C1—C2—C11'—C16'	−64.5 (7)
C2—S1—C3—N2	11.35 (19)	S1—C2—C11'—C16'	59.9 (7)
N2—N1—C4—C5	−177.36 (19)	C1—C2—C11'—C12'	109.3 (6)
N1—C4—C5—C6	174.4 (2)	S1—C2—C11'—C12'	−126.3 (5)
N1—C4—C5—C10	−4.2 (4)	C16'—C11'—C12'—C13'	2.2 (9)
C10—C5—C6—C7	0.8 (4)	C2—C11'—C12'—C13'	−171.6 (5)
C4—C5—C6—C7	−177.9 (2)	C11'—C12'—C13'—C14'	−0.9 (9)
C5—C6—C7—C8	1.2 (4)	C12'—C13'—C14'—C15'	−1.0 (13)
C6—C7—C8—F1	179.3 (2)	C13'—C14'—C15'—C16'	1.5 (16)
C6—C7—C8—C9	−2.3 (4)	C12'—C11'—C16'—C15'	−1.7 (11)
F1—C8—C9—C10	179.6 (2)	C2—C11'—C16'—C15'	172.2 (6)
C7—C8—C9—C10	1.1 (4)	C14'—C15'—C16'—C11'	−0.2 (14)
C8—C9—C10—C5	1.0 (4)		

### Hydrogen-bond geometry (Å, º)

**Table:**

*D*—H···*A*	*D*—H	H···*A*	*D*···*A*	*D*—H···*A*
N3—H3*A*···Br1	0.90	2.36	3.2557 (18)	172
N3—H3*B*···Br1^i^	0.90	2.55	3.3552 (18)	150
C4—H4*A*···Br1^ii^	0.93	2.99	3.726 (2)	137

Symmetry codes: (i) −*x*+1, −*y*, −*z*+1; (ii) *x*+1, *y*+1, *z*.
